# Clinicopathological and prognostic significance of NM23 expression in patients with non-small cell lung cancer: A systematic review and meta-analysis

**DOI:** 10.1097/MD.0000000000027919

**Published:** 2021-11-24

**Authors:** Shi-hui Min, Qiang-qiang Zheng

**Affiliations:** a Department of Oncology, West China School of Public Health and West China Fourth Hospital, Sichuan University, Chengdu, Sichuan, P.R. China; b Department of Thoracic Surgery, West China School of Public Health and West China Fourth Hospital, Sichuan University, Chengdu, Sichuan, P.R. China.

**Keywords:** clinicopathological features, meta-analysis, non-metastasis 23, non-small cell lung cancer, prognostic significance

## Abstract

**Background:**

There is a heated debate on the clinicopathological features and prognostic significance with non-metastasis 23 (NM23) expression in patients with non-small cell lung cancer (NSCLC). Thus, we conducted this meta-analysis to evaluate the clinicopathological features and prognostic significance of NM23 for NSCLC patients.

**Methods:**

Pubmed, Embase, and Web of Science were exhaustively searched to identify relevant studies published prior to March, 2020. Odds radios (ORs) and hazard radios with 95% confidence intervals (CIs) were calculated to summarize the statistics of clinicopathological and prognostic assessments. Q-test and I^2^-statistic were utilized to assess heterogeneity across the included studies. We also performed subgroup analyses and meta-regression analyses to identify the source of heterogeneity. Publication bias was detected by Begg and Egger tests. Sensitivity analysis was used to value the stability of our results. All the data were analyzed using statistical packages implemented in R version 4.0.5.

**Results:**

Data from a total of 3170 patients from 36 studies were extracted. The meta-analysis revealed that low expression of NM23 was correlated with higher risk of NSCLC (OR = 4.35; 95% CI: 2.76–6.85; *P* < .01), poorer tumor node metastasis (TNM) staging (OR = 1.39; 95% CI: 1.01–1.90; *P* = .04), poorer differentiation degree (OR = 1.37; 95% CI: 1.01–1.86; *P* = .04), positive lymph node metastasis (OR = 1.83; 95% CI: 1.22–2.74; *P* < .01), lung adenocarcinoma (OR = 1.45; 95% CI: 1.20–1.75; *P* < .01), and poorer 5-year overall survival (OS) rate (hazard radio = 2.33; 95%CI: 1.32–4.11; *P* < .01). The subgroup analyses and meta-regression analyses suggested that the “Publication year”, “Country”, “Sample size”, and “Cutoff value” might be the source of heterogeneity in TNM staging, differentiation degree, and lymph node metastasis. Both Begg test and Egger test verified that there were publication bias in 5-year OS rate. Sensitivity analysis supported the credibility of the results.

**Conclusion:**

The reduced NM23 expression is strongly associated with higher risk of NSCLC, higher TNM staging, poorer differentiation degree, positive lymph node metastasis, lung adenocarcinoma, and poorer 5-year OS rate in NSCLC patients, which indicated that NM23 could serve as a biomarker predicating the clinicopathological and prognostic significance of NSCLC.

## 1 Introduction

Lung cancer is the most frequently diagnosed cancer and the leading cause of cancer-related mortality for both men and women.^[1,2]^ Non-small cell lung cancer (NSCLC) approximately accounts for 85% of lung cancer and is associated with poor prognosis.^[2,3]^ Currently, advanced surgical techniques, anesthetic techniques, and perioperative management have significantly improved the safety of surgery, but the prognosis remains to be dismal and the 5-year overall survival (OS) rate approximates 15%,^[4]^ which is due to the complexity and heterogeneity of its progression and response to treatment. Therefore, early diagnosis of NSCLC still is the most significant strategy, and it is crucial to identify prognostic biomarker for NSCLC and provide clinical treatment strategies for patients with NSCLC.

Non-metastasis 23 (NM23) was initially found in a mouse tumor model by Steeg in 1988 and was the first discovered metastasis suppressor,^[5]^ and could encode a multifunctional protein that exerts anti-metastatic properties.^[6]^ In human cells, the NM23 gene is located on chromosome 17q21 that encodes a 17kDa protein with 152 amino acids, the 2 most considerable of the NM23 gene family are NM23-H1 and NM23-H2, which encode the A and B subunits of nucleoside diphosphate kinase (NDPK).^[7]^ NM23 transfers the γ-phosphate from nucleoside triphosphates to nucleoside diphosphates by forming a high energy phospho-histidine intermediate, and it has the role of NDPK, histidine kinase, and 3′-5′ exonuclease.^[8–10]^ The credible evidences have identified that NM23 do play a significant role in both cellular and extracellular processes including proliferation, embryonic development, differentiation, gene regulation, apoptosis, metastasis, DNA binding, DNA cleavage, and epithelial cell integrity.^[11–13]^

Owing to the role of enzymatic and kinase activities including acting as a NDPK, histidine/aspartic acid-specific protein kinase and serine protein kinase,^[14,15]^ the altered expression of NM23 protein may affect the function of cells. It has been indicated that downregulation of NM23 is associated with aggressive behavior in many types of tumors, but the exact mechanisms of metastasis suppression by NM23 remain a mystery. According to the current studies, NM23 maybe play a role via inhibiting the activities of important signaling pathways, which are necessary in the process of tumor invasion, such as Ras/mitogen-activited protein kinase and transforming growth factor-β,^[9,16]^ inhibiting the expression of matrix metalloproteinase-2 and suppressing the cloning ability of carcinoma cells.^[17,18]^ Thus, some authors thought that altered expression of NM23 is related to tumor invasion and metastasis. Indeed, it has been widely observed that altered expression of NM23 is associated with prognosis and clinicopathological features in several types of tumors, including breast cancer, ovarian cancer, cervical cancer, gastric cancer, and colorectal cancer.^[19–23]^

However, some controversies still exist on the relationship between NM23 expression with clinicopathological features and prognostic significance in NSCLC. On one hand, several evidences showed that reduced NM23 expression was linked with higher risk of NSCLC, higher tumor node metastasis (TNM) staging, poorer differentiation grade, positive lymph node metastasis, and poorer 5-year OS in NSCLC patients.^[24,25]^ On the other hand, a few studies found no correlation between NM23 expression with clinicopathological features and prognostic significance in NSCLC patients.^[26–28]^ To systematically evaluate the value of NM23 in NSCLC, we performed a meta-analysis to identify the relationship between NM23 expression with clinicopathological features and prognostic significance in patients with NSCLC.

## 2 Materials and methods

### 2.1 Database search strategy

We performed systematic literature search of PubMed, Embase (via Ovid interface), and Web of Science (via campus network of Sichuan University) from their incipiency to March, 2020. The following search terms were used: “lung cancer”, “lung neoplasm”, “lung tumor”, “lung adenocarcinoma”, “LUAD”, “lung squamous cell carcinoma”, “LUSC”, “NSCLC”, “pulmonary cancer”, “pulmonary neoplasm”, “pulmonary tumor”, “pulmonary adenocarcinoma”, “NM23”, “non-metastasis 23”, “NDPK”, “NME1”, and “NME2”. In addition, we also manually screened the reference of each article to obtain potential articles relevant to this review. Only studies published in English or Chinese were included. All the initially identified articles were scrutinized independently by 2 reviewers.

### 2.2 Study selection criteria

Two reviewers reviewed all candidate articles independently. Eligible studies were selected according to the following inclusion criteria: all patients must have NSCLC diagnosis conformed by pathology; immunohistochemistry (IHC) was applied for NM23 staining in surgical specimens; the studies contained adequate information about the association between NM23 expression with clinicopathological characteristics and 5-year OS rate in patients with NSCLC; and if the study population was duplicated, only the most complete or most recent report would be enrolled. The major exclusion criteria were as follow: the following articles were immediately excluded: meta-analyses, letters, reviews, editorial materials, meeting abstracts, case reports and expert opinions; not human studies; not related to research topics; and studies without adequate information about clinicopathological and prognostic characteristics.

### 2.3 Data extraction

Data were extracted from the selected studies by 2 independent investigators. The following information were extracted: publication data including first author, publication year, country, language, sample size, investigating category, detecting method, cutoff definition, and follow-up duration; clinicopathological characteristics, including TNM staging, the expression level of NM23 in NSCLC tissues and para-carcinoma tissues, the expression level of NM23 in lung adenocarcinoma (LUAD) tissues and lung squamous cell carcinoma tissues, differentiation degree, and lymph node metastasis; method to detect NM23 expression and number of patients stratified by NM23 expression; and clinical outcomes, including OS and its correlative hazard radios (HRs) with 95% confidence intervals (CIs), which were estimated from original articles or Kaplan-Meier survival curves.

### 2.4 Assessment of methodological quality and risk of bias

The Cochrane Risk of Bias (RoB) assessment tool^[29]^ and the Physiotherapy Evidence Database (PEDro) scale^[30]^ were used to assess the RoB and methodological quality of the trials included in the meta-analysis. Methodological quality and RoB were independently assessed by 2 authors.

The RoB evaluated the selection bias, performance bias, detection bias, attrition bias, reporting bias, and other bias.^[29]^ Each item was classified as low-risk, high-risk, or unclear according to the Cochrane Collaboration's tool.^[29]^ The PEDro score evaluated the methodological quality of a trial by assessing the random/concealed allocation, between-groups similarity at baseline, participant/therapist/assessor blinding, dropouts, intention-to-treat analysis, between-groups comparison, point measures, and variability data.^[30]^ A trial was considered of high quality when the PEDro score was ≥6 out of 10 points.

### 2.5 Data synthesis and analysis

To assess the relationship between NM23 expression with clinicopathological characteristics of patients with NSCLC, we determined odds radios (ORs) with 95% CIs as the appropriate summarized statistics. To assess the prognostic value of NM23 expression in NSCLC, HRs with 95% CIs served as the summarized estimates. In general, ORs and HRs could be extracted from the demographics or statistics which were reported in original articles. If no original statistic was reported, we extrapolated the HRs with 95% CIs from the survival data according to the method described by Tierney et al.^[31]^ If necessary, we could also extract the survival data by Engauge Digitizer 4.1 from the Kaplan-Meier survival curve. Heterogeneity across the studies was evaluated with Cochran *Q* statistic and Higgins *I*^2^ statistic.^[32]^ Random-effects model was used when significant heterogeneity existed among studies (*P* < .1 or I^2^ > 50%). Otherwise, a fixed-effects model was employed. Subgroup analyses and meta-regression analyses were conducted to explore potential cause of heterogeneity. Potential publication bias was analyzed using Begg funnel plot and Egger linear regression tests.^[33]^ The stability of the results in the included studies was assessed by a sensitivity analysis. All statistical tests were two-tailed with a *P* < .05 being considered statistically significant. All the data were analyzed using statistical packages implemented in R version 4.0.5, run in RStudio version 1.2.5042.

## 3 Results

### 3.1 Literature search

**Figure 1:**
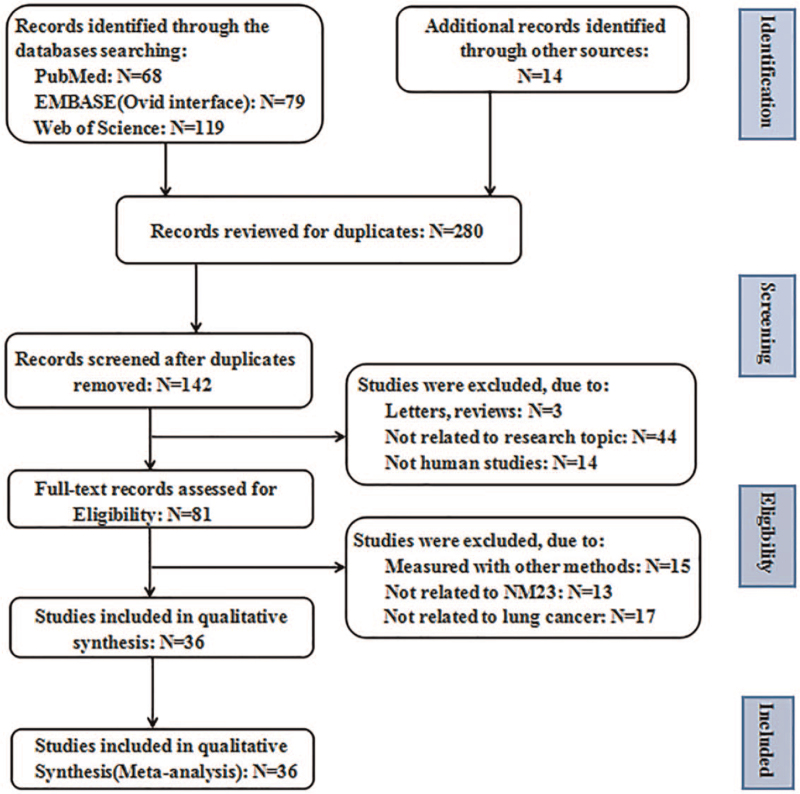
Flowchart of the study selection process.

Figure 1 presents the study selection process. Computer-based database searches and complementary manual search retrieved a total of 280 relevant articles. After removing 138 duplicates, we read the titles and abstracts of the 142 studies left, 61 studies were excluded because they either did not relate to research topic (n=44), or did not human studies (n = 14), or were letters and reviews (n = 3). After meticulously reading, 45 studies were excluded: 15 studies were measured with other methods, 13 studies were not related to NM23, and 17 studies were not related to lung cancer. As a result, 36 eligible studies^[24–28,34–64]^ with 3170 patients were enrolled in this meta-analysis.

### 3.2 Study characteristics

Table 1 shows the basic characteristics of the 36 studies, which were published from 1992 to 2015. Among them, 8 were published before 2000. Twenty-eight and 8 of the studies were carried out in China and foreign countries, respectively. The sample size ranged from 30 to 452 patients and a total of 3170 patients were included. All studies measured the NM23 expression in surgical specimens using IHC, and truncation values varied widely in all included studies.

**Table 1:**
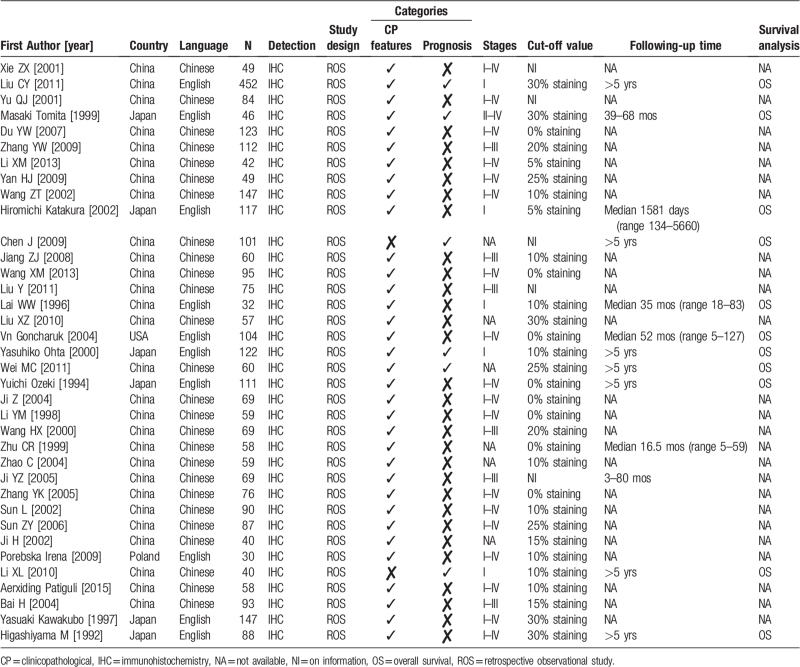
Main characteristics of included studies in this meta-analysis.

### 3.3 Risk of bias

**Figure 2:**
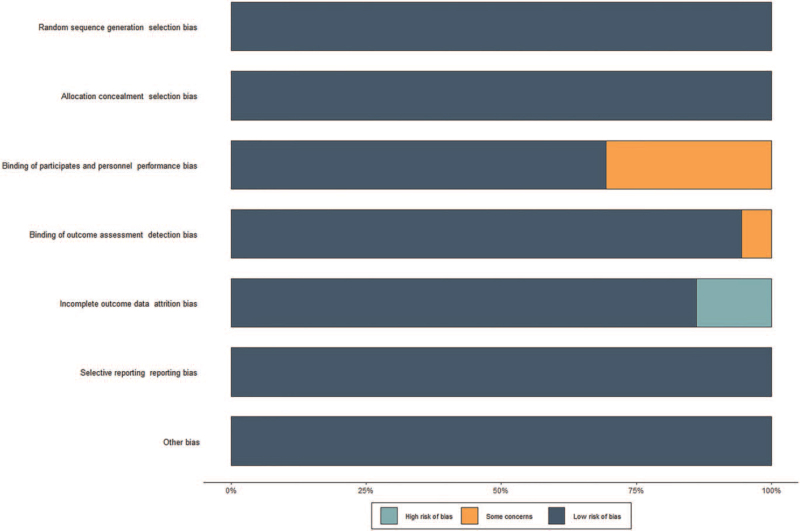
Plot of the risk of bias of the included studies.

RoB assessment of the included trials is summarized in Figure 2. No trial was able to blind therapists. In general, the RoB of the included trials in the current meta-analysis was low.

### 3.4 Relationship between NM23 expression with clinicopathological features in patients with NSCLC

**Figure 3:**
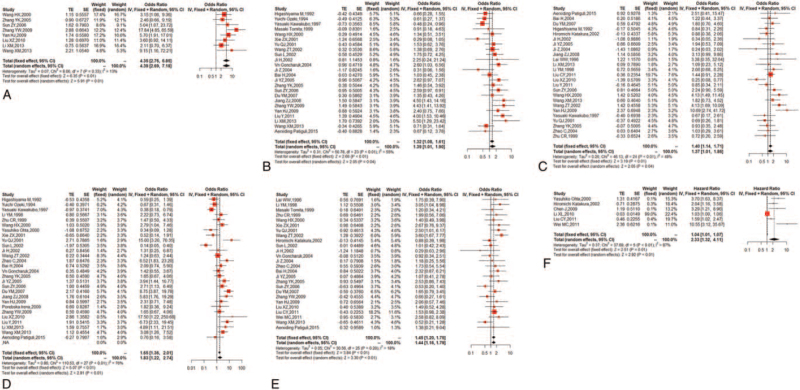
Forest plots demonstrating the effects of the NM23 on (A) risk of NSCLC, (B) TNM staging, (C) differentiation degree, (D) lymph node metastasis, (E) histotype, and (F) 5-year OS rate in patients with NSCLC. NM23 = non-metastasis 23, NSCLC = non-small cell lung cancer, OS = overall survival, TNM, tumor node metastasis.

The common clinicopathological parameters of NSCLC involved the risk of NSCLC, TNM staging, differentiation degree, lymph node metastasis, and histological subtypes in this meta-analysis. Pooled ORs and 95% CIs for NM23 expression, illustrated in Figure 3 and Table 2, revealed that reduced NM23 expression was significant associated with higher risk of NSCLC (OR = 4.35; 95% CI: 2.76–6.85; *P* < .01; I^2^ = 13%, *P* = .33) (Fig. 3A and Table 2), poorer TNM staging (OR = 1.39; 95% CI: 1.01–1.90; *P* = .04; I^2^ = 55%, *P* < .01) (Fig. 3B and Table 2), poorer differentiation degree (OR = 1.37; 95% CI: 1.01–1.86; *P* = .04; I^2^ = 48%, *P* < .01) (Fig. 3C and Table 2), positive lymph node metastasis (OR = 1.83; 95% CI: 1.22–2.74; *P* < .01; I^2^ = 76.2%, *P* < .01) (Fig. 3D and Table 2), and LUAD (OR = 1.45; 95% CI: 1.20–1.75; *P* < .01; I^2^ = 18%, *P* = .20) (Fig. 3E and Table 2).

**Table 2:** Meta-analysis for the association between NM23 expression with clinicopathological features and prognosis in patients with NSCLC.

					Publication bias	
Characteristics	N	Model	OR or HR (95% CI)	*P* value	Egger (*P*)	Conclusion
Risk of NSCLC (paracancerous tissues vs NSCLC tissues)	8	Fixed	4.35 (2.76–6.85)	<.01	0.3912	Significant
TNM staging (I/II vs III/IV)	25	Random	1.39 (1.01–1.90)	.04	0.3482	Significant
Differentiation degree (high/moderate vs low)	25	Random	1.37 (1.01–1.86)	.04	0.6963	Significant
Lymph node metastasis (N0 vs N1–3)	28	Random	1.83 (1.22–2.74)	<.01	0.1849	Significant
Histological subtypes (LUAD vs LUSC)	26	Fixed	1.45 (1.20–1.75)	<.01	0.8012	Significant
5-year OS rate (positive vs negative)	6	Fixed	2.33 (1.32–4.11)	<.01	0.0012	Significant

CI = confidence interval, HR = hazard ratio, LUAD = lung adenocarcinoma, LUSC = lung squamous cell carcinoma, NM23 = non-metastasis 23, NSCLC = non-small cell lung cancer, OR = odds ratio, OS = overall survival, TNM, tumor node metastasis.

### 3.5 Relationship between NM23 expression with prognosis in patients with NSCLC

There were 6 studies reporting the relationship of NM23 expression and 5-year OS rate in patients with NSCLC. As a result, the forest plot showed that reduced expression of NM23 was associated with poorer OS (HR = 2.33; 95%CI: 1.32–4.11; *P* < .01; I^2^ = 87%, *P* < .01) (Fig. 3F and Table 2).

### 3.6 Subgroup analyses

**Figure 4:**
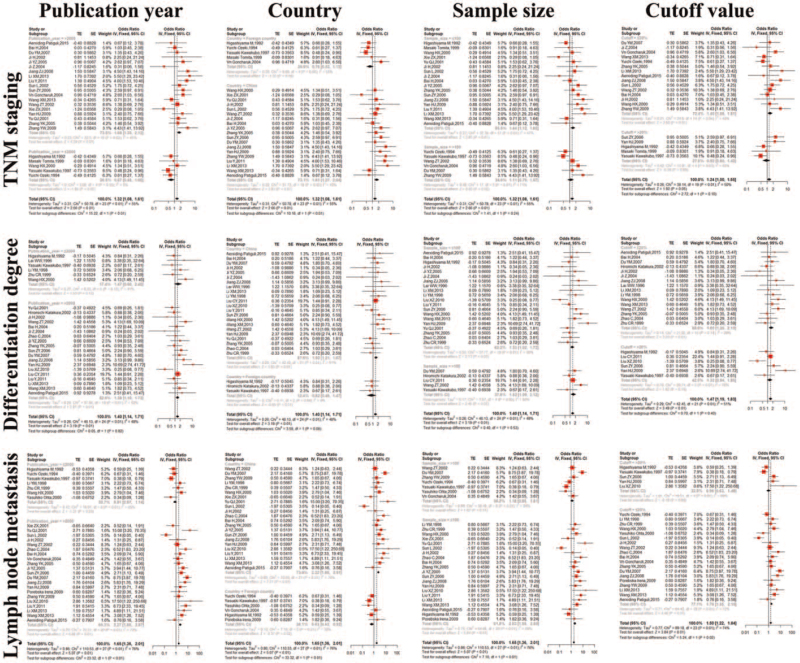
Forest plots of subgroup analyses for the association between NM23 expression with TNM staging, differentiation degree and lymph node metastasis in patients with NSCLC. NM23 = non-metastasis 23, NSCLC = non-small cell lung cancer, TNM, tumor node metastasis.

Because of significant heterogeneity, we conducted subgroup analyses in TNM staging, differentiation degree, and lymph node metastasis. We classified the articles into several subgroups according to publication year (≤2000 and >2000), country of patients (China and foreign country), sample size (≤100 and >100), and cutoff value (≤20% and >20%). In TNM staging, the subgroups of “Publication_year = >2000”, “Country = China” “Sample_size = ≤100”, and “Cutoff = ≤20%” had significant estimates (*P* < .01, *P* < .01, *P* < .01, *P* = .01, respectively) (Fig. 4 and Table 3). And with both of the “Publication_year = ≤2000” and “Publication_year = >2000” having I^2^ < 50%, the “Publication year” might be the source of heterogeneity of TNM staging. Although we obtained some meaningful data in subgroups of differentiation degree and lymph node metastasis, we could not find any possible source of heterogeneity in them (Fig. 4 and Table 3). Then we conducted meta-regression analyses to find the possible source of heterogeneity, and found that the “Publication year”, “Country”, “Sample size”, and “Cutoff value” might be the source of heterogeneity in TNM staging, differentiation degree, and lymph node metastasis because of the smaller Tau2.

**Table 3:** Subgroup meta-analysis for the association between NM23 expression with TNM staging, differentiation degree and lymph node metastasis in patients with NSCLC.

	TNM staging					Differentiation degree					Lymph node metastasis				
Factor	No.	OR (95% CI)	*P*	I^2^	Ph	No.	OR (95% CI)	*P*	I^2^	Ph	No.	OR (95% CI)	*P*	I^2^	Ph
All studies	24	1.32 (1.08–1.61)	<.01	55%	<0.01	25	1.40 (1.14–1.71)	<.01	48%	<0.01	28	1.65 (1.36–2.01)	<.01	76%	<0.01
Publication year															
≤2000	5	0.67 (0.45–1.00)	.05	0%	0.55	6	1.47 (0.90–2.40)	.13	41%	0.13	7	0.81 (0.57–1.14)	.23	65%	<0.01
>2000	19	1.68 (1.33–2.13)	<.01	45%	0.02	19	1.38 (1.10–1.73)	<.01	52%	<0.01	21	2.27 (1.80–2.87)	<.01	72%	<0.01
Country															
China	19	1.61 (1.27–2.04)	<.01	43%	0.02	22	1.50 (1.21–1.87)	<.01	51%	<0.01	22	2.32 (1.85–2.91)	<.01	70%	<0.01
Foreign country	5	0.76 (0.52–1.13)	.18	55%	0.06	3	0.82 (0.46–1.47)	.51	0%	0.94	6	0.63 (0.43–0.92)	.02	30%	0.21
Sample size															
≤100	17	1.44 (1.12–1.85)	<.01	47%	0.01	20	1.33 (1.02–1.72)	.03	50%	<0.01	21	2.03 (1.59–2.60)	<.01	69%	<0.01
>100	6	1.11 (0.79–1.57)	.55	71%	<0.01	5	1.52 (1.09–2.12)	<.01	50%	0.09	7	1.18 (0.86–1.61)	.31	84%	<0.01
Cutoff value															
≤20%	14	1.40 (1.08–1.81)	.01	43%	0.04	16	1.60 (1.20–2.13)	<.01	29%	0.14	19	1.70 (1.35–2.15)	<.01	72%	<0.01
>20%	6	0.92 (0.60–1.40)	.69	63%	0.03	6	1.32 (0.94–1.85)	.10	76%	<0.01	5	0.96 (0.63–1.48)	.86	80%	<0.01

CI = confidence interval, NM23 = non-metastasis 23, NSCLC = non-small cell lung cancer, OR = odds ratio, TNM, tumor node metastasis.

### 3.7 Publication bias

**Figure 5:**
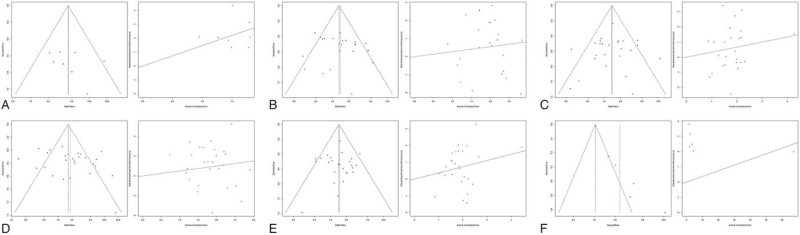
Funnel plots of NM23 for the clinicopathological features and prognosis. (A) Risk of NSCLC, (B) TNM staging, (C) differentiation degree, (D) lymph node metastasis, (E) histotype, (F) 5-year OS rate. NM23 = non-metastasis 23, NSCLC = non-small cell lung cancer, OS = overall survival, TNM, tumor node metastasis.

A funnel plot was used to discover the possible publication bias. Except the asymmetry of funnel plot in 5-year OS rate, the other plots were not obvious asymmetry (Fig. 5). In addition, the Egger tests revealed that there were no publication bias in risk of NSCLC (*P* = .3912), TNM staging (*P* = .3482), differentiation degree (*P* = .6963), lymph node metastasis (*P* = .1849), and histotype (*P* = .8012), but there were significant publication bias in 5-year OS rate (*P* = .0012) (Table 2).

### 3.8 Sensitivity analysis

**Figure 6:**
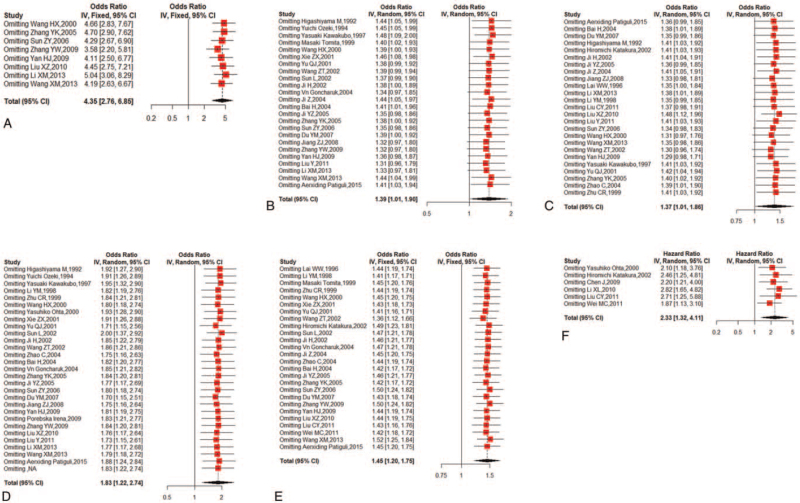
Sensitivity analysis for this meta-analysis. (A) Risk of NSCLC, (B) TNM staging, (C) differential grade, (D) lymph node metastasis, (E) histotype, (F) 5-year OS rate. NSCLC = non-small cell lung cancer, OS = overall survival, TNM, tumor node metastasis.

Sensitivity analysis was conducted to assess the stability of the studies and minimize the effect of individual research on conclusions. The included studies were sequentially omitted to assess whether any single study could have an impact on clinicopathological features and prognosis (Fig. 6). The sensitivity analysis suggested that the exclusion of any study did not alter the pooled results.

## 4 Discussion

NM23 is the first discovered metastasis suppressor gene, which does not affect the growth of primary tumor but a powerful inhibitor of metastases of tumors.^[23]^ Altered NM23 expression was found to be closely related to various tumor metastases, including NSCLC. However, it remains controversial about the relationship between NM23 expression with clinicopathological features and prognostic significance. In addition, no meta-analysis has previously been published on this topic. Hence, in this study, we conducted the first comprehensive systematic review and meta-analysis to figure out the actual effects of NM23 on patients with NSCLC.

In this meta-analysis, we included a total of 36 eligible studies involving a total of 3170 NSCLC patients. Our meta-analysis found that low NM23 expression was significantly correlated to elevated NSCLC risk, higher TNM staging, poorer tumor differential grade, and positive lymph node metastasis. Previous studies have found that elevated expression of NM23 protein was associated with the decreased metastatic capacity of many malignant tumors.^[6,65]^ The reduced NM23 protein expression contributes substantially to the metastatic process, indicating that metastatic tumor cells are mainly composed of cells with low NM23 expression. Data from previous studies and our meta-analysis showed that NM23 expression levels were correlated with the pathological characteristics of NSCLC, which strongly supported that NM23 expression was related to the carcinogenesis and progression of NSCLC.

Another crucial result in our study was that NM23 may be used as a prognostic indicator of NSCLC. We found that NM23-positive NSCLC patients had a higher 5-year OS rate than NM23-negative NSCLC patients, and the 5-year tumor metastasis rate in NM23-positive NSCLC patients was observed at a lower level than in NM23-negative NSCLC patients. Hence, NM23 is a positive indicator of favorable NSCLC prognosis. In conclusion, our results demonstrated that NM23 expression was associated with the progression of NSCLC, which is consistent with the report of You et al.^[66]^ Therefore, NM23 expression can be used as a reliable and independent prognostic indicator of NSCLC.

In this study, there were significant heterogeneity in TNM staging, differentiation degree, and lymph node metastasis, we conducted subgroup analyses to find out the source of heterogeneity subsequently. The subgroup analyses results suggested that the “Publication year” might be the source of heterogeneity in TNM staging. The meta-regression analyses subsequently identified that “Publication year”, “Country”, “Sample size”, and “Cutoff value” might be the source of heterogeneity in TNM staging, differentiation degree, and lymph node metastasis.

Although the NM23 is the first identified metastasis suppressor gene, its role in the growth of lung cancer cells and the exact molecular mechanism of NM23 inhibiting metastasis are still unclear. Previous studies have found that NM23 may play a role in inhibiting tumor metastasis through the following mechanisms. Firstly, loss of heterozygosity (LOH) and microsatellite instability of NM23 were 2 independent genetic pathways and crucial mechanisms in the carcinogenesis and progression of various of cancers, including esophageal squamous cell carcinoma, gallbladder tumor, gastric cancer, and colorectal cancer.^[67–70]^ It also has been identified that LOH and microsatellite instability of NM23 were associated with highly aggressive tumors and poor survival.^[67–70]^ In addition, the LOH rate of NM23 with metastasis was significantly higher than that without metastasis in human lung cancer cells.^[71]^ Secondly, NM23 could play a role via inhibiting the activities of important signaling pathways and controlling the expression of metastasis-related proteins. Some studies have shown that NM23-H1/H2 might exert its anti-metastatic effect through blockage of Ras/extracellular regulated protein kinases signaling.^[72,73]^ Boissan et al^[7]^ identified that the deficiency of NM23, via knockdowning NM23-H1 in hepatoma and colon carcinoma cell lines, increased expression of several pro-invasive signaling pathways such as the Akt and mitogen-activited protein kinase/stress-activated protein kinase pathways. In addition, the protein kinase A, Wnt, and protein kinase C signaling pathways have also demonstrated a connection to the effect of NM23.^[74–76]^ NM23 could also work through controlling the expression of metastasis-related proteins, including increasing expression of β-catenin, E-cadherin, and tissue inhibitor of metalloproteinases-1, and decreasing expression of matrix metalloproteinase-2, CD44 antigen variant 6, and vascular endothelial growth factor C.^[77]^ Finally, epithelial-mesenchymal transition (EMT) is a process through which epithelial cells undergo multiple biochemical changes to acquire mesenchymal phenotype and increase migratory capacity. NM23 has been reported to suppress transforming growth factor-β1-induced EMT,^[78]^ which maybe associated with the increased expression of E-cadherin.^[77]^ EMT results in the weaken of adhesion and enhance of migration of tumor cells, which makes tumor cells entering the blood and generates circulating tumor cells, and the circulating tumor cells are able to predict metastatic relapse and are related to disease progression and worse clinical outcome.^[79]^

Admittedly, several limitations existed in our meta-analysis. Firstly, some of the eligible studies had relatively small sample sizes. Secondly, we adopted articles written in English and Chinese, which would lose some available studies in other languages. Thirdly, all of the enrolled articles were non-randomized controlled trial (RCT) studies, and some bias, such as selection bias, misclassification bias, and information bias, might be present in this meta-analysis. Fourthly, the expression level of NM23 was detected by IHC, the studies investigated by other methods might affect the results of our meta-analysis. Fifthly, this meta-analysis based on semi-quantitative approaches which describe “increase” and “decrease” or “positive” and “negative” as measured factors, which was not adequately precise. Finally, we estimated the 5-year OS rate from Kaplan-Meier curves in some original articles, which might be less reliable than the data given by the original articles. Therefore, well-conducted RCTs exploring the effect of NM23 in patients with NSCLC are required.

## 5 Conclusions

We conducted the first meta-analysis to investigate the clinicopathological features and prognostic significance of NM23 for patients with NSCLC. We found that reduced NM23 expression was significantly correlated to higher NSCLC risk, higher TNM staging, poorer differentiation degree, positive lymphatic metastasis, LUAD, and poorer 5-year OS rate in NSCLC patients. NM23 may serve as a valuable biomarker in diagnosis and prognosis of NSCLC. Further well-designed RCTs are badly needed to confirm and update our conclusions.

## Author contributions

**Conceptualization:** Shi-hui Min, Qiang-qiang Zheng.

**Data curation:** Qiang-qiang Zheng.

**Formal analysis:** Shi-hui Min, Qiang-qiang Zheng.

**Methodology:** Shi-hui Min, Qiang-qiang Zheng.

**Supervision:** Shi-hui Min.

**Writing – original draft:** Shi-hui Min, Qiang-qiang Zheng.

**Writing – review & editing:** Shi-hui Min, Qiang-qiang Zheng.
